# Defense pattern of Chinese cork oak across latitudinal gradients: influences of ontogeny, herbivory, climate and soil nutrients

**DOI:** 10.1038/srep27269

**Published:** 2016-06-02

**Authors:** Xiao-Fei Wang, Jian-Feng Liu, Wen-Qiang Gao, Yun-Peng Deng, Yan-Yan Ni, Yi-Hua Xiao, Feng-Feng Kang, Qi Wang, Jing-Pin Lei, Ze-Ping Jiang

**Affiliations:** 1 State Key Laboratory of Tree Genetics and Breeding, Key Laboratory of Tree Breeding and Cultivation of State Forestry Administration, Research Institute of Forestry, Chinese Academy of Forestry, Beijing 100091, China; 2 The Research Institute of Tropical Forestry, Chinese Academy of Forestry, Guangzhou 510520, China; 3 College of Forestry, Beijing Forestry University, Beijing 100083, China; 4 Guangdong Key Laboratory of Agricultural Environment Pollution Integrated Control, Guangdong Institute of Eco-Environmental and Soil Sciences, Guangzhou 510650, China

## Abstract

Knowledge of latitudinal patterns in plant defense and herbivory is crucial for understanding the mechanisms that govern ecosystem functioning and for predicting their responses to climate change. Using a widely distributed species in East Asia, *Quercus variabilis*, we aim to reveal defense patterns of trees with respect to ontogeny along latitudinal gradients. Six leaf chemical (total phenolics and total condensed tannin concentrations) and physical (cellulose, hemicellulose, lignin and dry mass concentration) defensive traits as well as leaf herbivory (% leaf area loss) were investigated in natural Chinese cork oak (*Q. variabilis*) forests across two ontogenetic stages (juvenile and mature trees) along a ~14°-latitudinal gradient. Our results showed that juveniles had higher herbivory values and a higher concentration of leaf chemical defense substances compared with mature trees across the latitudinal gradient. In addition, chemical defense and herbivory in both ontogenetic stages decreased with increasing latitude, which supports the latitudinal herbivory-defense hypothesis and optimal defense theory. The identified trade-offs between chemical and physical defense were primarily determined by environmental variation associated with the latitudinal gradient, with the climatic factors (annual precipitation, minimum temperature of the coldest month) largely contributing to the latitudinal defense pattern in both juvenile and mature oak trees.

Knowledge of the latitudinal patterns in biotic interactions, especially herbivory, is crucial for understanding the mechanisms that govern ecosystem functioning and for predicting their responses to climate change^1^. Agrawal^2^ reported that leaf loss caused by insect herbivores account for more than 20% of net primary productivity, and an outbreak of such herbivores could even convert forests from net carbon sinks to net carbon sources^3^. Furthermore, the existing scenarios generally predict that increasing temperatures will cause a faster increase in herbivory than in plant productivity^4^,^5^. Population densities of plant-feeding insects are predicted to increase because of the direct beneficial effects of a warmer climate on insect herbivores^6^ and the adverse effects of an increase in climatic variability on their natural enemies^7^. These predictions are supported by the positive association noted between herbivory and the mean temperature of the growth season observed along latitudinal gradients outside the tropics^8^ and by palaeontological data that demonstrate enhanced herbivory with climatic warming in the past^9^. Therefore, climate is often considered to be the primary driver of latitudinal patterns in biotic interactions, and latitudinal gradients have recently been promoted as a natural laboratory for studying the potential impacts of a changing climate on terrestrial organisms^10^.

The prevailing hypothesis in plant latitudinal defense patterns is a low latitude/high defense hypothesis. This was generally regarded as being a result of winter mortality, which would tend to prevent insect herbivores from reaching density limitations of their populations at high latitudes, or as a result of co-evolution between plants and herbivores where high herbivore pressure often occurred at low latitudes. This hypothesis was supported by numerous studies^11^,^12^,^13^. For example, Pearse & Hipp analysed the defensive traits of 56 oak species using phylogenetic methods and found that oak leaf defenses were higher at lower latitudes or regions with a low temperature seasonality, mild winters, and a low minimum precipitation^12^. However, inconsistent or opposite trends were reported by other researchers^14^,^15^,^16^,^17^,^18^. The most impressive review, conducted by Moles *et al.*^15^, on 38 latitudinal comparisons of herbivory, showed that chemical defenses were significantly higher in plants from higher latitudes but with no significant effect of latitude on the physical defenses of plants, and suggesting that the available data do not completely support the low latitude/high defense hypothesis. Kim^19^ also found no relationship or feedback between plant defenses and herbivore pressure, thus suggesting that the latitudinal variation in plant resistance is complex and possibly constrained by resource availability and trade-offs in plant defenses. Moreover, most related studies have focused on the community level, whereas a comparison of the pattern of plant defenses and herbivory of a single species across latitudinal gradients has received less attention^20^.

Apart from the abiotic determinants of plant defenses, e.g., temperature^12^,^21^, precipitation^22^,^23^, and soil nutrient availability^24^,^25^, ontogenetic variation could play a crucial role in plant defense mechanisms^26^,^27^. As plants develop from seeds to seedlings and from juveniles to mature stages, their ontogeny can constrain the expression of resistance to herbivore damage. Nevertheless, ecological and evolutionary theories regarding interactions between plants, herbivores and their natural enemies are largely based on observations and experiments conducted at a single ontogenetic stage. Due to resource allocation and architectural constraints in plants, as well as the influence of herbivore foraging behaviour, resistance to herbivores likely changes during plant development^28^. Two prominent hypotheses suggest different mechanisms behind the ontogenetic patterns in plant defenses against herbivory, but both frequently result in conflicting predictions. The growth-differentiation-balance hypothesis (GDBH) emphasizes intrinsic factors, such as the acquisition and allocation of resources, which limit the production of defensive secondary compounds in young plants, thereby leading to an ontogenetic increase in defenses^29^. In contrast, the optimal-defense hypothesis (ODH) focuses on extrinsic factors, such as selection by herbivores, which lead to high levels of defenses in juveniles, followed by a decrease as plants mature^30^. Numerous studies testing the above hypotheses have documented both increases and decreases in plant defensive traits across ontogeny, offering partial support for both hypotheses (see review by Barton & Koricheva^26^). Nonetheless, how latitudinal defense patterns are affected by plant ontogeny remains poorly understood and may be of importance to further understanding the observed latitudinal patterns in plant defenses and herbivory.

In plant herbivore defenses, the trade-offs between chemical and physical defenses have been widely used to examine defense investments under various herbivory pressures from the perspective of resource allocation^14^,^31^,^32^. Total phenolics and tannin concentration are the main surrogates used for chemical defense^33^; whereas the three main components of plant cell wall fiber, i.e., cellulose, hemicellulose and lignin, which contribute to overall material strength^34^, have been widely used as surrogates for physical defense. Cornelissen^35^ identified a triangular relationship between the total phenolics and fibre across thirty-four subarctic species, i.e., species exhibited a high phenolics concentration with low fibre, or the inverse, or low but never high quantities of both total phenolics and fibre. However, positive or null correlations between physical and chemical defenses have also been reported in other studies^36^,^37^,^38^. Moles *et al.*^39^ suggested that the absence of clear trade-offs in previous research could be a result of these studies using a pairwise approach between individual physical and chemical defensive traits rather than integrating the suite of chemical and physical defenses employed by each species. Furthermore, most studies focused on a single site and did not consider ontogenetic and environmental variation across a broad geographical scale.

The Chinese cork oak (*Quercus variabilis* Blume) is one of the most widespread tree species in eastern Asia, covering the area from approximately 19°–42°N and 97°–140°E^40^. The distribution regions differ greatly in terms of climatic and edaphic conditions. The species is an excellent candidate for studying the ontogenetic variation of plant defenses along latitudinal gradients, as well as the trade-offs between the defensive traits and environmentally driven mechanisms at a single species level. In the present study, living leaves of juvenile and mature trees were collected from seven latitudinal sites in China, spanning ~14° latitude (Fig. 1). Our aims are to test the following: (1) whether there is a similar trend in herbivory and defensive traits between juvenile and mature trees across latitudes; (2) whether the trade-off between chemical and physical defenses occurs in both ontogenetic stages; and (3) whether the dominant factors (climatic, edaphic and herbivory) that potentially shape latitudinal defense patterns differ between ontogenetic stages. Our results could help to predict the impact of climate change on herbivory characteristics and defense strategies /patterns in plants.

## Results

### Ontogenetic variation in defensive traits across latitudinal gradients

Both ontogeny and latitude had significant effects on the leaf chemical defensive substances (total phenolics and total condensed tannin concentration), but significant interactive effects were only found for the total phenolics concentration (F = 4.737, P = 0.001) (Table 1). Compared with mature individuals, juveniles had a significantly higher concentration of total phenolics (155.55 ± 36.77 mg/g in mature trees and 172.72 ± 51.48 mg/g in juvenile trees) and condensed tannins (71.80 ± 29.01 mg/g in mature trees and 92.14 ± 23.89 mg/g in juvenile trees). The total phenolics and condensed tannin concentration in both juvenile and mature tree leaves decreased with an increase in latitude (Fig. 2).

Tree age had no significant effect on the physical defensive traits (cellulose, hemicellulose, lignin, and LDMC). However, latitude significantly influenced the physical defenses, with the exception of hemicellulose (F = 2.061, P = 0.083). No age-latitude interactions were found to affect the physical defensive traits (Table 2). There were no significant trends across latitudes in any of the physical defense substances, although the concentrations of leaf cellulose and hemicellulose exhibited a slight increasing trend with latitude, whereas lignin decreased with latitude (Fig. 3).

Herbivory (% leaf area loss) was significantly affected by tree age, latitude (F = 15.516, P < 0.001 for age and F = 149.437, P < 0.001 for latitude) and their interaction (F = 5.251, P < 0.001) (Table 1). The leaves of juvenile trees experienced significantly higher herbivory (% leaf area loss) than the mature trees (12.15 ± 8.18% for mature trees and 14.23 ± 11.04% for juvenile trees), especially at sites on the southern edge (e.g., Cili and Chengbu) (Fig. 4). However, only latitude significantly affected herbivory measured in terms of % damaged leaf number (F = 156.802, P < 0.001). Leaf herbivory of both juvenile and mature trees decreased significantly with an increase in latitude (Fig. 4). In particular, leaf herbivory (% leaf area loss) increased from 5.56% (northern edge site, Beijing) to 22.91% (southern edge site, Chengbu Hunan) and from 5.08% (Beijing) to 29.77% (Chengbu Hunan) for mature and juvenile trees, respectively. Correspondingly, leaf herbivory (% damaged leaf number) increased from 59.17% to 99.17% and from 63.00% to 98.67% for mature and juvenile trees, respectively.

### Correlations between chemical and physical defensive traits and herbivory

We also tested the potential trade-offs between chemical and physical defensive traits, using the first axis eigenvalues of a PCA on these traits (Fig. 5), as well as the correlations between these traits and herbivory (% leaf area loss) using partial correlation analysis. As shown in Table 2 and Fig. 5, there were significantly negative correlations, namely, trade-offs, between chemical and physical defensive traits (r = −0.550, P < 0.001). Furthermore, these trade-offs were primarily determined by environmental variation (latitudes) rather than ontogenetic stages (Table 2), based on the results obtained for the controlling factors, i.e., ontogeny, latitude or a combination of both. Furthermore, the chemical and physical defensive traits were significantly positively and negatively correlated, respectively, with herbivory (r = 0.576, P < 0.001 for chemical; r = −0.612, P < 0.001 for physical).

### Redundancy analysis of defensive traits and environmental factors

Redundancy analysis (RDA) showed that annual precipitation, minimum temperature of the coldest month and herbivory were relatively important factors that influenced the distribution of defensive substances (Fig. 6). Among the two RDAs performed on mature and juvenile trees, 49.34% of the total variation in the six defensive traits was explained by eight environmental variables. In the case of the environmental variables for the mature and juvenile trees, the first two axes explained 51.67% and 40.07%, respectively, of the variance across seven latitudinal gradients (Appendix S4).

## Discussion

### Ontogenetic differences in defensive traits

Chinese cork oak exhibited contrasting chemical defense differences between the two ontogenetic stages, the juveniles exhibited higher leaf total phenolics and condensed tannin concentration compared with the mature trees. Leaf total phenolics, including condensed tannins, are ubiquitous in plants and are broadly distributed within a variety of plant tissues and cells, in keeping with their defensive roles^41^,^42^. The optimal defense theory predicts natural selection will favour young plants as they are more highly defended than older plants. This is based on the assumption that the impact of herbivory on plant fitness decreases with plants age^43^,^44^,^45^. The observed higher concentrations of total phenolics and condensed tannins in the leaves of juvenile oaks support such predictions. Studies on birch^46^, aspen^47^ and *Eucalyptus*^48^ have also found negative relationships between juvenile and mature trees with respect to foliar phenolics.

In contrast to the chemical defensive traits, the leaf physical defensive traits (concentrations of cellulose, hemicellulose, lignin and LMDC) did not exhibit a dominant trend across ontogeny, suggesting that physical defense is less plastic or more conservative than chemical defense. High cellulose should be positively correlated with leaf fracture toughness because the high tensile strength of cellulose contributes to fracture toughness^49^. By contrast, a high lignin content should be more correlated with stiffness than with toughness because lignin is a complex polymer with strong covalent bonds^50^. Lignin is also known for its anti-fungal properties; pathogenic attacks often induce localized and systemic increases in lignin production^51^. No ontogenetic differences in leaf fracture toughness in the present study may partly result from the fact that we sampled approximately similar aged leaves (mature leaves) rather than different aged leaves from trees of two ontogenetic stages across latitudinal gradients. Our results are consistent with the study of Alvarez-Clare *et al.*^52^, who found that leaf fracture toughness did not show distinctively ontogenetic changes across species, whereas this was not true for the findings reported by Quintero *et al.*^53^. Ochoa-López *et al.*^54^ stated that different ontogenetic trajectories in plants expressing multiple defensive strategies could be a consequence of: (a) changes in fitness costs and/or benefits of each defensive trait at particular ontogenetic stages^43^; (b) differences in herbivore preferences^53^; and/or (c) genetic correlations and trade-offs among different defensive strategies^55^.

### Latitudinal trends in herbivory and defensive traits

Our results revealed that chemical defensive traits and herbivory in the leaves of both juvenile and mature oak trees decreased with increasing latitude, which is consistent with a number of studies^11^,^12^,^13^,^20^,^56^, supporting the latitudinal herbivory-defense hypothesis, i.e., that plants at low latitudes will be better defended against herbivory than at high latitudes. Opposite or inconsistent trends were also observed in other studies^14^,^15^,^16^,^17^,^18^. Discrepancies in plant latitudinal defense patterns may be attributed to autologous features^57^,^58^, dominating environmental factors^59^ and a variety of natural enemies^60^,^61^,^62^. Not all plant species are equally defended against herbivores, which is evidenced by the wide variation in the types of resistance traits and degrees of resistance among species. Johnson *et al.*^63^ suggested that this variation can be attributed to a combination of two phenomena. First, plant populations adapt to maximize their fitness to local biotic and abiotic environmental conditions. Second, evolutionary trade-offs prevent populations from maximally defending themselves against all herbivores in every environment. These trade-offs can involve traits that affect growth, competition, tolerance to stress, mutualistic interactions and defense against natural enemies.

Trade-offs between chemical and physical defense were found for Chinese cork oak in the present study, which agrees with the findings of Eichenberg *et al.*^64^ but disagrees with the results of Cárdenas *et al.*^32^. These inconsistent results may arise from relatively homogeneous habitats and phylogenetic differences among tree species, which could obscure the trends. In the present study, there was no ontogenetic effect, but the strong latitudinal effect on the trade-offs between chemical and physical defense suggests the existence of these trade-offs depends more on environmental factors, such as temperature. In other words, environmental variation associated with latitude could influence a plant allocation or investment into chemical and physical defenses differently.

### Relationship between environmental factors and defensive traits of oak

Generally, environmental factors should affect plant growth and development, as well as the synthesis and distribution of secondary metabolites of organisms. For example, a meta-analysis performed by Ushio & Adams^65^ revealed that annual mean temperature (AMT) was positively correlated with the abundance of foliar condensed tannins, while annual precipitation (AP) and latitude did not have a significant influence on the concentration of condensed tannins. Additionally, Hallam & Read^66^ reported that total phenolics and tannin concentration increased with increasing AMT. However, we did not find significant correlations between chemical defensive substances (e.g., total phenolics, condensed tannin concentration) and AMT. Our results revealed that the defensive traits of oak were primarily determined by meteorological factors (MTCM and AP) and herbivory rather than AMT. In our sampling regions in China (beyond the tropics), we found that AMT was not significantly or positively associated with decreasing latitude. However, MTCM and AP had a better linear relationship with latitude, and both the extreme coldest and warmest temperature occurred in the northern region. There are two possible explanations for this finding, namely, the dual effects of climatic factors. On the one hand, the extreme climates of higher latitudes are commonly believed to restrict the breeding (survival and reproduction) of insects or herbivores^56^,^67^, resulting in lower insect density and consequently less leaf damage at higher latitudes. Similarly, moisture is also an influential factor. Coley & Barone^68^ found that at the community level, leaf herbivory also varied under different moisture environments, with higher rates in a wet forest (48.0%) than in a dry forest (14.2%). Accordingly, abundant hydrothermal conditions could enhance herbivore activity, which could result in higher herbivore pressure. Furthermore, the potential higher herbivore pressure could induce plants to generate more defensive substances to enhance their anti-herbivore ability. On the other hand, in the absence of herbivory, meteorological factors could directly affect the concentrations of defensive substances in plants. For example, ultraviolet stress could induced increases in some secondary metabolites, e.g., phenols and flavonoids^69^,^70^, which act as phytoalexin or sunscreen to avoid damage from abiotic factors (ultraviolet radiation). Furthermore, Close & MacArthur^70^ suggested that UV light flux increases by approximately 1–2% per 1° latitude towards the equator. Therefore, across a large-scale latitudinal gradient, ultraviolet radiation may be an important factor in the variation of phenolic compounds and should be considered when addressing related issues.

However, other studies found no clear relationships between the concentration of phenolic compounds and air temperature or latitude^14^,^71^. One possible explanation for these contradictory results is that some plant species may have other defensive strategies that are independent of the direct carbon-based chemical defensive substances (such as total phenolics and condensed tannins). For example, Heil *et al.*^72^ proposed a protective ant-plant interaction theory and emphasized that ants could effectively protect plants. Consequently, ant-associated plants have a lower amount of direct defensive substrates compared with those with a lower intensity of ant-defenses^73^,^74^. Additionally, redundancy analysis confirmed that edaphic factors were less important than meteorological factors and herbivory, and the correlations with defensive traits were not clear in the ordination pattern for Chinese cork oak at a regional scale. It is believed that, at a local spatial scale, defensive substances (for example, foliar condensed tannin concentration) vary widely depending on the soil nutrient availability and developmental stage of the plants^65^,^75^.

## Conclusion

In conclusion, the findings of this study showed that both the chemical defensive traits (i.e., total phenolics, condensed tannin concentration) and herbivory of leaves of oak trees decreased with increasing latitude. The leaves of juvenile trees had higher total phenolics and condensed tannin concentrations compared with those of mature trees. Additionally, we found that there were trade-offs between the chemical and physical defenses of Chinese cork oak. Finally, annual precipitation, the minimum temperature of the coldest month and herbivory were decisive factors influencing the distribution of the defensive substances of the studied species. One limitation of this study is that only seven latitudinal sites were sampled. To our knowledge, however, this is the first study to compare ontogenetic differences in defenses along a latitudinal gradient. The genetic variation in oak defenses as well as some specific meteorological factors, i.e., ultraviolet radiation, which were not included in our analysis, should be considered in future studies. Moreover, the latitudinal pattern of plant defense need be further tested and investigated in future studies by employing more widely distributed species to provide an integrated analysis of the complex interactions between plant defensive traits, abiotic and biotic factors, and herbivory. Although soil conditions had less effect on the plant defense characteristics in the present study, the mechanism and effect of soil nutrients on the chemical synthesis of defense substances in plants should be further explored and analysed using manipulative experiments in future studies.

## Methods

### Sampling strategies

**Figure 1:**
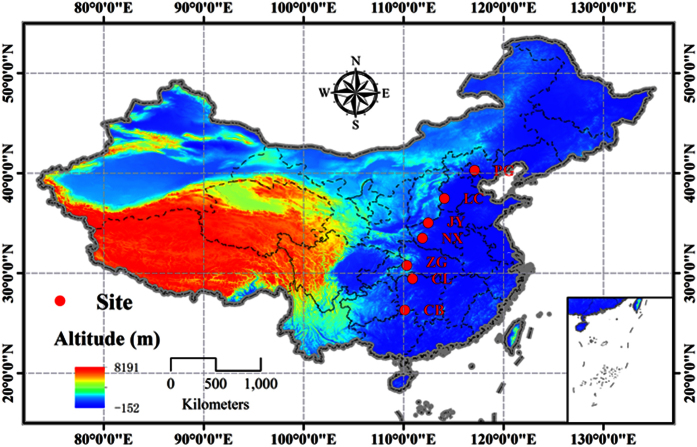
Map of the *Quercus variabilis* sampling sites in China (PG: Pinggu, Beijing; LC: Lincheng, Hebei; JY: Jiyuan, Henan; NX: Neixiang, Henan; ZG: Zigui, Hubei; CL: Cili, Hunan and CB: Chengbu, Hunan). The map was drawn by the software using ArcGIS (v.10.0), http://www.esri.com/. *Scientific Reports* remains neutral with regard to contested jurisdictional claims in published maps.

**Figure 2:**
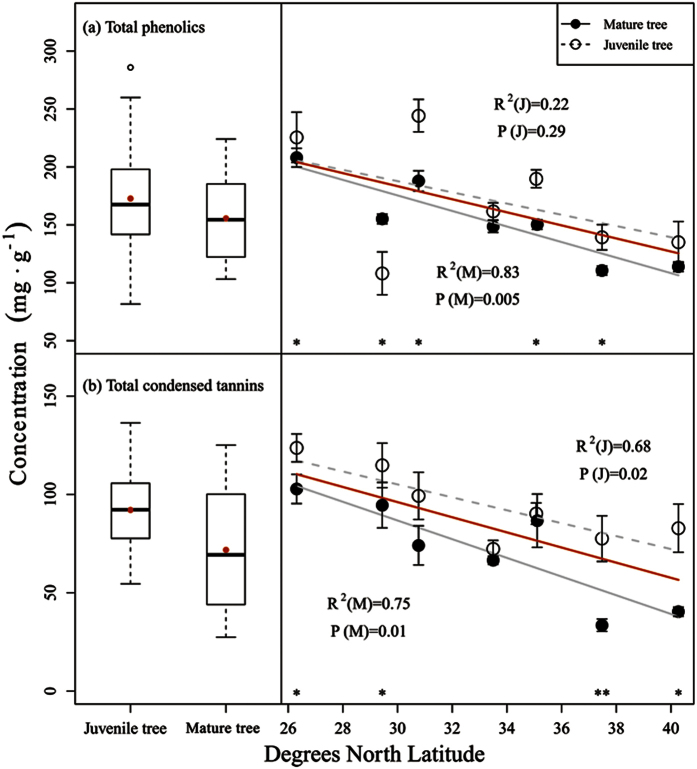
Total phenolics (**a**) and total condensed tannin (**b**) concentration (mean ± sd) in *Quercus variabilis* leaves at two ontogenetic stages versus site latitude. The total condensed tannin concentration is the sum of extractable CT, protein-bound CT and fibre-bound CT (see S1). The *R*^2^ coefficients and *P* values shown were obtained using simple linear regression models. Each point represents the mean at a certain latitudinal site (a total of 4 plots were established at each latitudinal site; three juvenile and three mature trees were chosen per plot for sampling purposes). The error bars represent the standard deviation of the average value. Asterisks represent significant differences at P < 0.05 (*) and P < 0.01 (**) based on a T-test.

**Figure 3:**
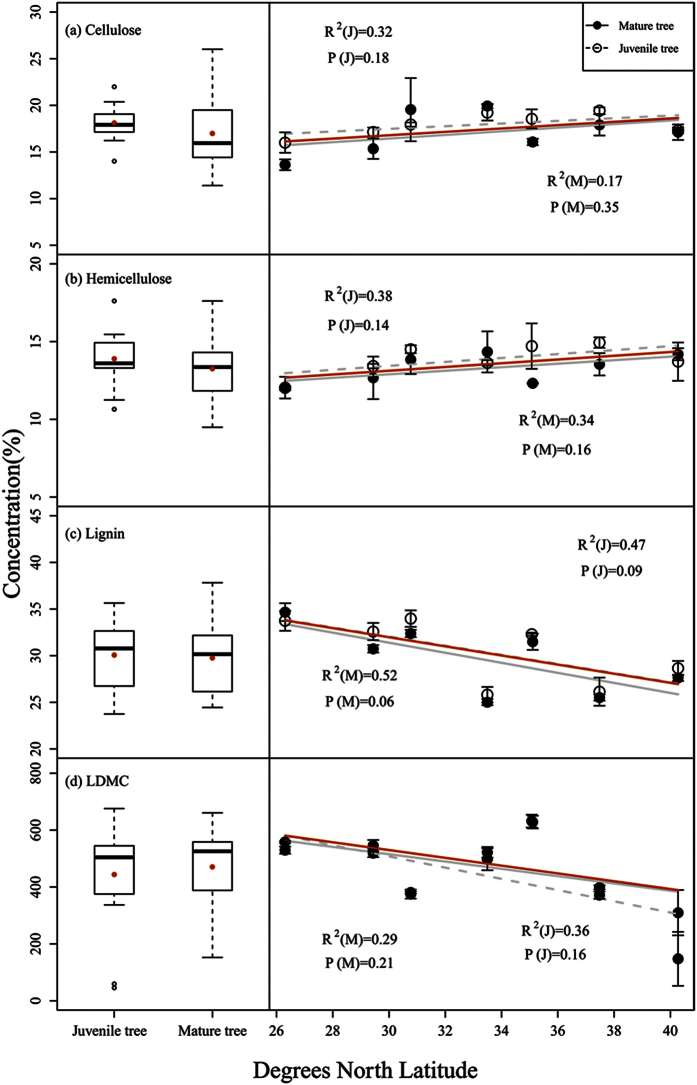
Cellulose, hemicellulose, lignin and LDMC concentrations (mean ± sd) in *Quercus variabilis* leaves at two ontogenetic stages versus latitude. (**a–d**) are cellulose, hemicellulose, lignin and LDMC, respectively. The *R*^2^ coefficients and *P* values shown were obtained using simple linear regression models. Each point represents the mean of a certain latitudinal site (a total of 4 plots were established at each latitudinal site; three juvenile and three mature trees were chosen per plot for sampling purposes). The error bars represent the standard deviation of the average value. Asterisks represent significant differences at P < 0.05 (*) and P < 0.01 (**) based on a T-test.

**Figure 4:**
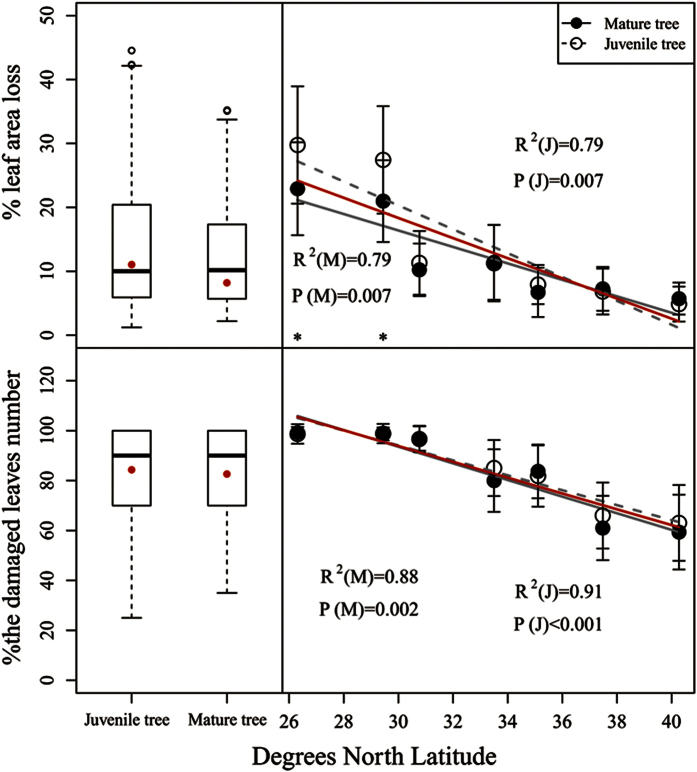
Herbivory (%, percentage of leaf area loss and percentage of damaged leaf number) (mean ± sd) across the latitudinal gradient. 40.27, Pinggu, Beijing; 37.48, Lincheng, Hebei; 35.06, Jiyuan, Henan; 33.50, Neixiang, Henan; 30.77, Zigui, Hubei; 29.45, Cili, Hunan; and 26.30, Chengbu, Hunan. The *R*^2^ coefficients and *P* values shown were obtained using simple linear regression models. Each point represents the mean at a certain latitudinal site (a total of 4 plots were established at each latitudinal site; three juvenile and three mature trees were chosen per plot for sampling purposes). Approximately 900 replicates (leaves) per site were used for the assessment of herbivory. Asterisks represent significant differences at P < 0.05 (*) based on a T-test.

**Figure 5:**
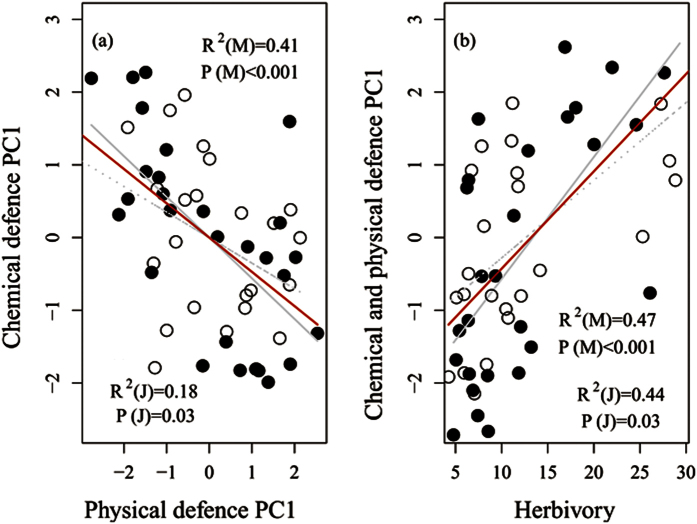
Relationships between chemical (TP and TCT) and physical defensive traits (cellulose, hemicellulose, lignin and LDMC) (**a**) and between defensive traits (including chemical and physical) and the herbivory (% leaf area loss) (**b**) in the oak tree leaves. Significant negative relationships (P < 0.05) denote a trade-off between chemical and physical defenses. Each point represents the mean value of three juvenile and/or three mature trees from one sample plot. Solid circles (●): mature trees; Empty circles (○): juvenile trees; solid line (—) is fitted line of mature trees; dashed line (- -) is fitted line of juvenile trees.

**Figure 6:**
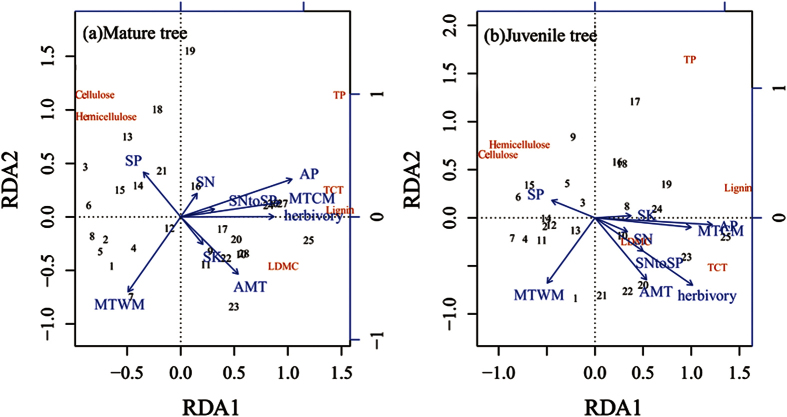
Redundancy analysis results showing the relationships among chemical and physical defensive traits and environmental variables. The variables are listed as follows: TP, total phenolics; TCT, total condensed tannin concentration; cellulose, cellulose concentration; hemicellulose, hemicellulose concentration; lignin, lignin concentration; LDMC, leaf dry mass content; SN, SP, SK, soil N, P, K concentration; SNtoSP, soil N:P ratio; herbivory; AMT, annual mean temperature; MTWM, maximum temperature of the warmest month; MTCM, minimum temperature of the coldest month; and AP, annual precipitation.

Because the concentrations in plant defensive substances differed between phenological stages^76^, our sampling times were first estimated using Gong & Jian’s method^77^ to avoid the phenological differences caused by latitudinal gradients (Table 3). The sampling tasks were performed from south to north during the middle of the growing season (August, 2014), covering a total of 7 sites spanning a 14°- latitudinal gradient (~26°–40°N) (Fig. 1, Table 3). At each site, four 50 × 50 m temporary plots were established, with three mature trees and three juvenile trees in each plot and a total of 24 individuals were selected (Table 4). The mature trees were estimated to be approximately 100~150 years old with a range in diameter at breast height (1.3 m above ground, DBH) from 15 to 20 cm, and the juvenile trees were approximately 7~10 years old with a range in DBH from 3 to 6 cm.

We collected healthy and well-developed leaves with no sign of herbivory or pathogen damage from the mature and juvenile trees (leaves were collected from the upper middle parts of branches exposed to direct/full sunlight without shading by neighbouring trees to ensure consistent light conditions). Thus, a total of 168 leaf samples (4 plots × 7 sites × 2 ontogenetic stages × 3 replicate individuals) were selected and immediately stored in an ice box. In the laboratory, the leaves from the same individual were divided into two parts: one for the determination of the cellulose, hemicellulose and lignin concentrations (dried at 105 °C for 0.5 h, then 70 °C for 48 h) and the other for the determination of the total phenolics and tannin concentration (dried in the shade for 45 days in a drying room with a relative humidity of 10%), according to published methods^78^. All samples were ground and sieved through an 80-mesh sieve (0.20 mm diameter) for further chemical analysis.

### Herbivory assessment

The herbivore damage to leaf was assessed by measuring the leaf areas. The selected individuals were the same as above. Leaves were randomly selected from five branches per plot and scanned using a flatbed scanner (Leaf Area Meter, Yaxin-1241, Beijing, China) to evaluate herbivory (including % leaf area loss and the % damaged leaf number). The calculation formulas^14^,^79^ are as follows:


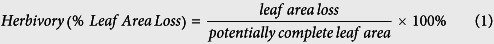



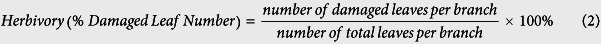


### Chemical analysis

#### Chemical defensive traits

Total phenolic (TP) concentrations (mg/g) were determined using the Folin-Ciocalteu phenol method^80^ with gallic acid as the standard. The absorbance was read at 765 nm using a DU800 Spectrophotometer (Beckman Coulter, Fullerton, CA). The total condensed tannin (TCT) concentration was calculated as the sum of extractable condensed tannins (ECT) and bound condensed tannins (BCT), where BCT was the sum of protein-bound CT (PBCT) and fibre-bound CT (FBCT). ECT and BCT were assayed using the butanol/HCl method^81^,^82^ with procyanidins as the standard. All chemicals were of analytical reagent (AR) purity grade. The TP standard (gallic acid) and CT standard (procyanidins) were purchased from Sigma Chemical Co. (St. Louis, MO). The detailed procedure is described in Appendix S1.

#### Physical defensive traits

The fibre concentrations of dried and ground leaves were determined using standard biomass analytical methods (“NREL”, the National Renewable Energy Laboratory) and separated by HPLC (LC-20AT, Shimadzu, Japan). The powder was enclosed in a chemical resistant bag, and treated with a series of increasingly aggressive extractants to i) determine the oven dry weight (ODW) of the extractive-free sample, using the average total solids concentration as determined by the LAP “Standard Method for the Determination of Total Solids in Biomass”, and to ii) calculate the amount of acid insoluble residue (AIR), acid insoluble lignin (AIL) and acid soluble lignin (ASL)^83^. From these data, hemicellulose, cellulose and lignin were calculated by subtraction (the detailed procedure is shown in Appendix S2). The LDMC was calculated as the proportion of leaf dry mass per leaf fresh mass.

### Soil data

We collected three soil samples (30 cm in depth) from each plot and pooled them as one replicate. The soil samples were air-dried and ground to pass through a 2-mm sieve after stones and plant materials were removed. Then, the soil samples were digested in a solution of H_2_SO_4_–HClO_4_, and then the N concentrations (SN, %) were determined using a Kjeltec analyser (Kjeltec 2300 Analyzer Unit, Sweden). The P concentrations (SP, %) were colorimetrically determined using blue phosphor-molybdate (6505 UV spectrophotometer, UK), and the K concentrations (SK, %) using flame photometry (ZL-5100 atomic absorption spectrophotometer, USA)^84^. All chemical determinations were repeated three times using the same subsamples. The results are presented on mass basis (%).

### Climatic data

The monthly climatic data (2014) were used to explore climate effects on the concentrations of plant defensive substances; these data were interpolated (Kriging method) from 675 national weather stations around China using GIS software (ArcGIS v10.0, ESRI, USA). The specific values at each sampled site were then extracted according to the geolocation information (latitude and longitude) of the site. The climatic variables include the annual mean temperature (AMT, °C), temperature seasonality (TS, expressed as the standard deviation of temperature among months * 100), maximum temperature of the warmest month (MTWM, °C), minimum temperature of the coldest month (MTCM, °C), annual precipitation (AP, mm), precipitation of the wettest month (PWM, mm), precipitation of the driest month (PDM, mm), and the precipitation seasonality (PS, expressed as the coefficient of variation in precipitation across months).

### Statistical analyses

All graphical and data analyses were conducted using the R statistics package (version 3.0.1, http://www. r-project.org/). Each sampling site was considered an experimental unit with 3 or 4 replicates (plots). Simple linear regression models were performed to investigate whether latitude and climate were related to chemical defensive substances (total phenolics and total condensed tannin concentration) and physical defensive traits (cellulose, hemicellulose, lignin and LDMC). On all graphs where detected values are presented, the mean values and the corresponding standard errors of the mean are provided for each site. Two-way analysis of variance (ANOVA) was also performed to determine the statistical significances of the chemical and physical defensive traits between ontogenetic stages and latitudinal sites (Table 2).

**Figure 7:**
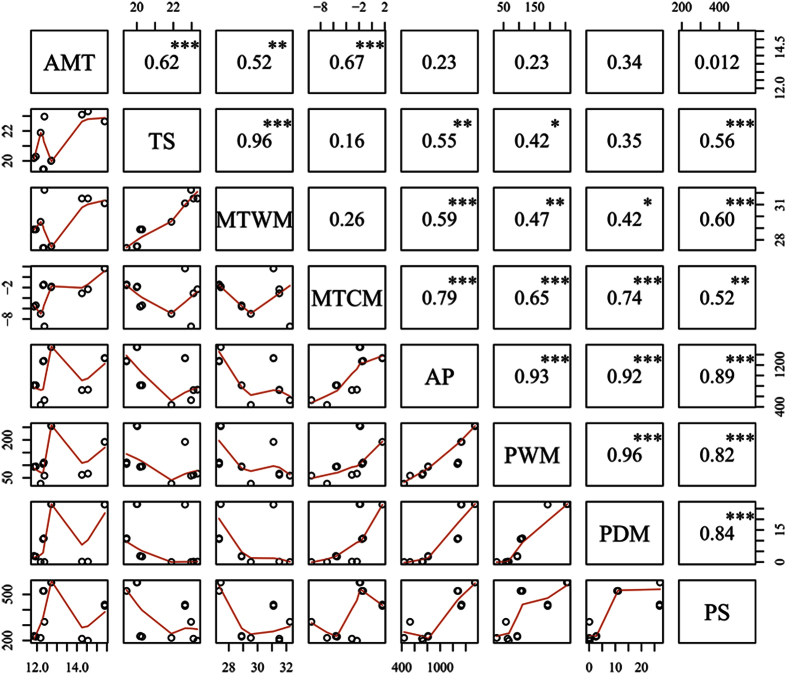
Correlation analysis among eight meteorological variables in 2014. The variables are listed as follows: AMT (annual mean temperature, °C); TS (temperature seasonality, expressed as the standard deviation of temperature among months * 100); MTWM (maximum temperature of the warmest month, °C); MTCM (minimum temperature of the coldest month, °C); AP (annual precipitation, mm); PWM (precipitation of the wettest month, mm); PDM (precipitation of the driest month, mm); and PS (precipitation seasonality, expressed as the coefficient of the variation in precipitation across months). Only significant effects are shown: *P < 0.05; **P < 0.01; ***P < 0.001.

To identify the interrelationships between the dependent variables (measured parameters) and independent variables (environmental data) and to obtain a direct gradient ordination, a redundancy analysis (RDA)^85^ was performed. The dependent variables included six defensive traits of oak leaves, i.e., total phenolics, total condensed tannins, cellulose, hemicellulose, lignin, and LDMC. The independent variables included: (1) eight climatic variables; (2) four soil variables, i.e., total nitrogen, total phosphorous, total potassium and total N/P ratio, and (3) herbivory. According to the correlation analysis among the eight meteorological variables (Fig. 7), there was a highly significant correlation between the TS (temperature seasonality) and MTWM (maximum temperature of the warmest month) (r = 0.96, P < 0.001), and between AP, PWM, PDM and PS (r > 0.9, P < 0.001). Therefore, only AMT, MTWM, MTCM and AP were selected for redundancy analysis to explore the relationship between environmental factors and defensive traits.

## Additional Information

**How to cite this article**: Wang, X.-F. *et al.* Defense pattern of Chinese cork oak across latitudinal gradients: influences of ontogeny, herbivory, climate and soil nutrients. *Sci. Rep.* **6**, 27269; doi: 10.1038/srep27269 (2016).

## Supplementary Material

Supplementary Information

## Figures and Tables

**Table 1:** Summary of two-way ANOVA results of chemical and physical defensive traits and herbivory among two ontogenetic stages and seven latitudinal sites.

		Ontogenetic stage	Latitude	Ontogenetic stage × Latitude
Total phenolics	*df*	1	6	6
	*F*	9.905	31.228	4.737
	*P*	0.003**	<0.001***	0.001**
Total condensed tannin	*df*	1	6	6
	*F*	19.156	13.160	1.631
	*P*	<0.001***	<0.001***	0.168
Cellulose	*df*	1	6	6
	*F*	2.977	4.112	0.859
	*P*	0.093	0.003**	0.534
Hemicellulose	*df*	1	6	6
	*F*	2.742	2.061	0.943
	*P*	0.107	0.083	0.477
Lignin	*df*	1	6	6
	*F*	0.418	47.939	0.558
	*P*	0.522	<0.001***	0.760
LDMC	*df*	1	6	6
	*F*	2.292	27.911	1.538
	*P*	0.139	<0.001***	0.195
Herbivory (% leaf area loss)	*df*	1	6	6
	*F*	15.516	149.437	5.251
	*P*	<0.001***	<0.001***	<0.001***
Herbivory (% damaged leaf number)	*df*	1	6	6
	*F*	2.867	156.802	1.251
	*P*	0.0912	<0.001***	0.279

Significant effects are shown: ***P < 0.001; **P < 0.01; *P < 0.05.

**Table 2:** Partial correlation analysis (Pearson, two-tailed test) under various control factors.

Control factor	Variables	ChemPC1	PhysPC1	Herbivory
None	ChemPC1		−0.550***	0.576***
	PhysPC1			−0.612***
	Ontogeny	0.000	0.000	−0.045
	Latitude	−0.789***	0.613***	−0.840***
Ontogeny	ChemPC1		−0.550***	0.576***
	PhysPC1			−0.613***
Latitude	ChemPC1		−0.137	−0.262 (P = 0.061)
	PhysPC1			−0.227
Ontogeny & Latitude	ChemPC1		−0.135	−0.274 (P = 0.052)
	PhysPC1			−0.224

Note: Significance level: ***P < 0.001; **P < 0.01; *P < 0.05.

**Table 3:** The geographic characteristics of the *Quercus variabilis* sampling sites.

Sites	Latitude	Longitude	Altitude /m	slope /°	slope direction	canopy density	Approximate Date at same phenological phase
Pinggu, Beijing	N40°16′	E117°07′	229~328	19~31	S, SW	0.6~0.8	20^th^ August
Lincheng, Hebei	N37°28′	E114°05′	646~702	15~42	SW	0.6~0.7	14^th^ August
Jiyuan, Henan	N35°01′	E112°28′	373~450	17~33	SW, SE	0.6~0.7	9^th^ August
Neixiang, Henan	N33°29′	E111°54′	861~960	19~42	S, SW, SE	0.6~0.8	6^th^ August
Zigui, Hubei	N30°46′	E110°20′	874~1034	6~18	SW	0.4~0.5	1^st^ August
Cili, Hunan	N29°26′	E110°54′	558~697	5~12	SE, SW	0.6~0.8	26^th^ July
Chengbu, Hunan	N26°18′	E110°07′	968~1220	20~45	S, SW	0.6~0.7	22^th^ July

The sampling date is estimated by using Gong & Jian’s (1983) method in terms of biological activity starting from a “standard” date of August 20 in Pinggu, Beijing at 40°N.

**Table 4:** Description of the diameter at breast height (DBH, cm) and height (m) of the sampled trees.

Sites	Mature trees		Juvenile trees	
	DBH	Height	DBH	Height
Pinggu, Beijing	17.48 ± 2.48	9.17 ± 2.11	3.86 ± 2.40	4.64 ± 1.67
Lincheng, Hebei	19.98 ± 3.69	10.79 ± 1.80	5.38 ± 0.37	5.48 ± 1.57
Jiyuan, Henan	18.08 ± 1.69	9.99 ± 0.99	5.34 ± 0.13	4.74 ± 2.22
Neixiang, Henan	17.84 ± 1.54	11.09 ± 1.25	4.80 ± 0.56	5.33 ± 1.71
Zigui, Hubei	18.10 ± 1.92	11.27 ± 1.32	5.25 ± 0.71	5.58 ± 1.86
Cili, Hunan	15.42 ± 3.45	9.35 ± 0.94	5.21 ± 0.20	5.98 ± 2.30
Chengbu, Hunan	17.56 ± 2.81	9.20 ± 1.60	5.36 ± 0.04	4.98 ± 1.20

A total of 4 plots were established at each latitudinal site, where three juveniles and three mature trees were sampled in each plot. Data show the mean value ± sd.
