# Supplementary material for: Defense pattern of Chinese cork oak across latitudinal gradients: influences of ontogeny, herbivory, climate and soil nutrients
Source: Sci Rep. 2016 Jun 2;6:27269. doi: 10.1038/srep27269 (PMC4890039; doi:10.1038/srep27269)
Supplement: Supplementary Information [file srep27269-s1.doc]

**Supplementary Information**

**Title: Defense pattern of Chinese cork oak across latitudinal gradients: influences of ontogeny, herbivory, climate and soil nutrients**

**Authors: Xiao-Fei Wang^1^, Jian-Feng Liu^1,*^, Wen-Qiang Gao^1^, Yun-Peng Deng^1^, Yan-Yan Ni^1^, Yi-Hua Xiao^2^, Feng-Feng Kang^3^, Qi Wang^4^, Jing-Pin Lei^1^, and Ze-Ping Jiang^1^**

**Appendix**

Appendix S1 Total condensed tannin (TCT) analysis

Appendix S2 Procedure for firbe content analysis

Appendix S3 The total condensed tannin (TCT) components concentration in leaves of the mature and juvenile *Quercus variabilis.* A, Pinggu; B, Lincheng; C, Jiyuan; D, Neixiang; E, Zigui; F, Cili; G, Chengbu. Total condensed tannin (TCT) was sum of extractable condensed tannins (ECT) and bound condensed tannins (BCT), where BCT was sum of protein-bound CT (PBCT) and fibre-bound CT (FBCT).

Appendix S4. Results of a Redundancy Analysis summarizing the information of eight independent climatic descriptors across all sites. Factor loadings, Eigen values and % of variance explained of the two main principal components (RDA1 and RDA2) are shown. Values in bold show factor loadings greater than 0.5.

Appendix S5 The meteorological data of the sampling sites of *Quercus variabilis*

**Appendix S1 Total condensed tannin (TCT) analysis**

Total condensed tannin (TCT) was sum of extractable condensed tannins (ECT) and bound condensed tannins (BCT), where BCT was sum of protein-bound CT (PBCT) and fibre-bound CT (FBCT). ECT and BCT were assayed using the butanol/HCl method (Terrill et al. 1992; Lin et al. 2006) with procyanidins as the standards. The absorbance at 550 nm was read on a DU800 Spectrophotometer (Beckman Coulter).

Extractable condensed tannins (ECT)

After removing a 5 ml of the pooled extracts for total phenolics analysis, the remainder of the extract was adjusted to 0.001 M ascorbic acid to minimize oxidation.The aqueous fractions were further analyzed by butanol/HCl assay (Terrill et al. 1992).

Protein-bound condensed tannin (PBCT)

To extract protein-bound CT as Terrill et al.(1992) described, 15 ml SDS solution (10 g·L^-1^ SDS and 50 g·L^-1^ 2-mercaptoethanol in 10 mM Tris/chloride buffer, pH 8.0) was added to pellet 1 from the above centrifugation. The tubes were shaken on a vortex mixer at 100℃ for 45 min, after which they were cooled to room temperature, centrifuged at 5000 r/min for 15 min, and the supernatant was poured into another 50 ml conical flask. The pellet was reextracted three times and the supernatants combined. Protein-bound condensed tannins were assayed by the butanol/HCl method (Terrill et al. 1992).

Fibre-bound condensed tannin (FBCT)

Fibre-bound CT was determined directly on the residue remaining from the extraction of protein-bound CT. The pellet was washed into a 50 ml glass centrifuge tube with 3.0 ml SDS solution, 21.0 ml butanol/HCl solution was added and maintained at 100℃ for 75 min. Tubes were cooled on ice, centrifuged at 5000r/min for 15 min, and the absorbance at 550 nm was read on a DU800 Spectrophotometer.

**Appendix S2 Procedure for firbe content analysis**

**Prepare the sample for analysis and hydrolyze**

Place an appropriate number of filtering crucibles in the muffle furnace at 575±25°C for a minimum of four hours. Remove the crucibles from the furnace directly into a desiccator and cool for a specific period of time, one hour is recommended. Weigh the crucibles to the nearest 0.1 mg and record this weight. It is important to keep the crucibles in a specified order, if they are not marked with identifiers. Permanent marking decals are available from Wale Apparatus. Do not mark the bottom of the filtering crucible with a porcelain marker, as this will impede filtration.

Place the crucible back into the muffle furnace at 575±25°C and ash to constant weight. Constant weight is defined as less than±0.3 mg change in the weight upon one hour of re-heating the crucible.

Weigh 300.0±10.0 mg of the sample or QA standard into a tared pressure tube. Record the weight to the nearest 0.1 mg. Label the pressure tube with a permanent marker. LAP “Determination of Total Solids in Biomass” should be performed at the same time, to accurately measure the percent solids for correction. Each sample should be analyzed in duplicate, at minimum. The recommended batch size is three to six samples and a QA standard, all run in duplicate.

Add 3.00±0.01 mL of 72% sulfuric acid to each pressure tube. Use a Teflon stir rod to mix for one minute, or until the sample is thoroughly mixed.

Place the pressure tube in a water bath set at 30±3°C and incubate the sample for 60±5 minutes. Using the stir rod, stir the sample every five to ten minutes without removing the sample from the bath. Stirring is essential to ensure even acid to particle contact and uniform hydrolysis.

Upon completion of the 60-minute hydrolysis, remove the tubes from the water bath. Dilute the acid to a 4% concentration by adding 84.00±0.04 mL deionized water using an automatic burette. Dilution can also be done by adding 84.00±0.04 g of purified water using a balance accurate to 0.01 g. Screw the Teflon caps on securely. Mix the sample by inverting the tube several times to eliminate phase separation between high and low concentration acid layers.

Prepare a set of sugar recovery standards (SRS) that will be taken through the remaining hydrolysis and used to correct for losses due to destruction of sugars during dilute acid hydrolysis. SRS should include D-(+)glucose, D-(+)xylose, D-(+)galactose, -L(+)arabinose, and D-(+)mannose. SRS sugar concentrations should be chosen to most closely resemble the concentrations of sugars in the test sample. Weigh out the required amounts of each sugar, to the nearest 0.1 mg, and add 10.0 mL deionized water. Add 348 µL of 72% sulfuric acid. Transfer the SRS to a pressure tube and cap tightly.

A fresh SRS is not required for every analysis. A large batch of sugar recovery standards may be produced, filtered through 0.2 µm filters, dispensed in 10.0 mL aliquots into sealed containers, and labeled. They may be stored in a freezer and removed when needed. Thaw and vortex the frozen SRS prior to use. If frozen SRS are used, the appropriate amount of acid must be added to the thawed sample and vortexed prior to transferring to a pressure tube.

Place the tubes in an autoclave safe rack, and place the rack in the autoclave. Autoclave the sealed samples and sugar recovery standards for one hour at 121°C, usually the liquids setting. After completion of the autoclave cycle, allow the hydrolyzates to slowly cool to near room temperature before removing the caps.

**Analyze the sample for acid insoluble lignin**

Vacuum filter the autoclaved hydrolysis solution through one of the previously weighed filtering crucibles. Capture the filtrate in a filtering flask.

Transfer an aliquot, approximately 50 mL, into a sample storage bottle. This sample will be used to determine acid soluble lignin as well as carbohydrates, and acetyl if necessary. Acid soluble lignin determination must be done within six hours of hydrolysis. If the hydrolysis liquor must be stored, it should be stored in a refrigerator for a maximum of two weeks.(a)

Use deionized water to quantatively transfer all remaining solids out of the pressure tube into the filtering crucible. Rinse the solids with a minimum of 50 mL fresh deionized water. Hot deionized water may be used in place of room temperature water to decrease the filtration time.

Dry the crucible and acid insoluble residue at 105±3 °C until a constant weight is achieved, usually a minimum of four hours.

Remove the samples from the oven and cool in a desiccator. Record the weight of the crucible and dry residue to the nearest 0.1 mg.

Place the crucibles and residue in the muffle furnace at 575±25 °C for 24±6 hours.

Carefully remove the crucible from the furnace directly into a desiccator and cool for a specific amount of time, equal to the initial cool time of the crucibles. Weigh the crucibles and ash to the nearest 0.1 mg and record the weight. Place the crucibles back in the furnace and ash to a constant weight. (The amount of acid insoluble ash is not equal to the total amount of ash in the biomass sample. Refer to LAP “Determination of Ash in Biomass” if total ash is to be determined.)

**Analyze the sample for acid soluble lignin**

On a UV-Visible spectrophotometer, run a background of deionized water or 4% sulfuric acid.

Using the hydrolysis liquor aliquot obtained in step (a), measure the absorbance of the sample at an appropriate wavelength on a UV-Visible spectrophotometer. Refer to section11.3 for suggested wavelength values. Dilute the sample as necessary to bring the absorbance into the range of 0.7 – 1.0, recording the dilution. Deionized water or 4% sulfuric acid may be used to dilute the sample, but the same solvent should be used as a blank. Record the absorbance to three decimal places. Reproducibility should be±0.05 absorbance units. Analyze each sample in duplicate, at minimum. (This step must be done within six hours of hydrolysis.)

**Analyze the sample for structural carbohydrates**

Prepare a series of calibration standards containing the compounds that are to be quantified, referring to Table 1 for suggested concentration range. Use a four point calibration. If standards are prepared outside of the suggested ranges, the new range for these calibration curves must be validated.

Prepare an independent calibration verification standard (CVS) for each set of calibration standards. Use reagents from a source or lot other than that used in preparing the calibration standards. Prepare the CVS at a concentration that falls in the middle of the validated range of the calibration curve. The CVS should be analyzed on the HPLC after each calibration set and at regular intervals throughout the sequence, bracketing groups of samples. The CVS is used to verify the quality and stability of the calibration curve(s) throughout the run.

Using the hydrolysis liquor obtained in step (a), transfer an approximately 20 mL aliquot of each liquor to a 50 mL Erlenmeyer flask.

Use calcium carbonate to neutralize each sample to pH 5 – 6. Avoid neutralizing to a pH greater that 6 by monitoring with pH paper. Add the calcium carbonate slowly after reaching a pH of 4. Swirl the sample frequently. After reaching pH 5 – 6, stop calcium carbonate addition, allow the sample to settle, and decant off the supernatant. The pH of the liquid after settling will be approximately 7. (Samples should never be allowed to exceed a pH of 9, as this will result in a loss of sugars.)

Prepare the sample for HPLC analysis by passing the decanted liquid through a 0.2 µm filter into an autosampler vial. Seal and label the vial. Prepare each sample in duplicate, reserving one of the duplicates for analysis later if necessary. If necessary, neutralized samples may be stored in the refrigerator for three or four days. After this time, the samples should be considered compromised due to potential microbial growth. After cold storage, check the samples for the presence of a precipitate. Samples containing a precipitate should be refiltered, while still cold, through a 0.2 µm filters.

Analyze the calibration standards, CVS, and samples by HPLC using a Shodex sugar SP0810 or Biorad Aminex HPX-87P column equipped with the appropriate guard column.

HPLC conditions:

Injection volume: 50 µL, dependent on concentration and detector limits

Mobile phase: HPLC grade water, 0.2 µm filtered and degassed

Flow rate: 0.6 mL / minute

Column temperature:85°C

Detector temperature: as close to column temperature as possible

Detector: refractive index

Check test sample chromatograms for presence of cellobiose and oligomeric sugars. Levels of cellobiose greater than 3 mg/mL indicate incomplete hydrolysis. Fresh samples should be hydrolyzed and analyzed.

Check test sample chromatograms for the presence of peaks eluting before cellobiose (retention time of 4-5 minutes using recommended conditions). These peaks may indicate high levels of sugar degradations products in the previous sample, which is indicative of over hydrolysis. All samples from batches showing evidence of overhydrolysis should have fresh samples hydrolyzed and analyzed.

**Appendix S3 The total condensed tannin (TCT) components concentration in leaves of the mature and juvenile *Quercus variabilis.* A, Pinggu; B, Lincheng; C, Jiyuan; D, Neixiang; E, Zigui; F, Cili; G, Chengbu. Total condensed tannin (TCT) was sum of extractable condensed tannins (ECT) and bound condensed tannins (BCT), where BCT was sum of protein-bound CT (PBCT) and fibre-bound CT (FBCT).**

**Appendix S4. Results of a Redundancy Analysis summarizing the information of eight independent climatic descriptors across all sites. Factor loadings, eigen values and % of variance explained of the two main principal components (RDA1 and RDA2) are shown. Values in bold show factor loadings greater than 0.5.**

| Defensive traits | Mature trees | |  | Juvenile trees | |
| --- | --- | --- | --- | --- | --- |
|  | PC1 | PC2 |  | PC1 | PC2 |
| Annual mean temperature | 0.4119 | **-0.6815** |  | 0.4498 | 0.2145 |
| Maximum temperature of the warmest month | -0.4457 | -0.3058 |  | -0.3458 | -0.2608 |
| Minimum temperature of the coldest month | **0.7591** | -0.5294 |  | **0.7777** | 0.4724 |
| Annual precipitation | **0.8874** | -0.0605 |  | **0.9347** | 0.1563 |
| Soil N content | 0.1787 | **0.5957** |  | 0.2567 | -0.4349 |
| Soil K content | 0.1494 | -0.2063 |  | 0.2813 | 0.1575 |
| Soil P content | -0.2815 | 0.2031 |  | -0.3456 | 0.0984 |
| Soil N/P ratio | 0.2976 | 0.2146 |  | 0.3953 | -0.3372 |
| Herbivory | **0.5660** | -0.1417 |  | **0.7322** | -0.0994 |
| Eigen value | 2.9313 | 0.6858 |  | 1.8896 | 0.9155 |
| % Variance Explained | 41.88 | 9.80 |  | 26.99 | 13.08 |
| % Cumulative propartion | 41.88 | 51.67 |  | 26.99 | 40.07 |

**Appendix S5 The meteorological data of the sampling sites of *Quercus variabilis***

| Sites | 2014 | | | |  | 1970-2000 | | | | |
| --- | --- | --- | --- | --- | --- | --- | --- | --- | --- | --- |
|  | AMT | MTWM | MTCM | AP |  | AMT | MTWM | MTCM | AP | RH |
| Pinggu, Beijing | 12.37 | 32.25 | -9.41 | 529.12 |  | 10.19 | 29.8 | -12 | 591.71 | 57.76 |
| Lincheng, Hebei | 12.19 | 29.53 | -7.01 | 435.68 |  | 9.88 | 28.27 | -9.8 | 535.56 | 60.06 |
| Jiyuan, Henan | 14.25 | 31.51 | -3.11 | 717.69 |  | 12.56 | 30.04 | -5.89 | 642.8 | 62.65 |
| Neixiang, Henan | 11.86 | 28.9 | -5.62 | 814.3 |  | 11.04 | 27.69 | -5.12 | 899.88 | 68.48 |
| Zigui, Hubei | 12.34 | 27.32 | -1.38 | 1282.12 |  | 9.88 | 24.2 | -3.47 | 1643.03 | 81.48 |
| Cili, Hunan | 15.39 | 31.1 | 1.64 | 1335.34 |  | 14.41 | 30.15 | 0.03 | 1490.57 | 79.28 |
| Chengbu, Hunan | 12.71 | 27.44 | -1.93 | 1542.53 |  | 12.79 | 27.26 | -0.44 | 1603.3 | 82.79 |

Note: AMT (annual mean temperature, °C), MTWM (maximum temperature of the warmest month, °C), MTCM (minimum temperature of the coldest month, °C), AP (annual precipitation, mm). RH(relative humidity, %). we used a subset of the bioclimatic variables (available at <http://www.ncdc.noaa.gov/>). Climate data in unobserved locations was estimated by interpolating (Kriging method) among the climate station locations.
